# Brugada syndrome during physical therapy: a case report

**DOI:** 10.1186/1757-1626-1-107

**Published:** 2008-08-18

**Authors:** Jan Paul M Frölke, Almar WA Bruggeman, Frank PAJ Klomp, Joep LRM Smeets

**Affiliations:** 1 Dept. of Surgery 690, University Hospital Nijmegen, P.O. Box 9101, 6500 HB, Nijmegen, The Netherlands; 2 Dept. of Surgery, Section Traumatology, 690, University Hospital Nijmegen, P.O. Box 9101, 6500 HB, Nijmegen, The Netherlands; 3 Dept. of Physical Therapy 645, University Hospital Nijmegen, P.O. Box 9101, 6500 HB, Nijmegen, The Netherlands; 4 Dept. of Cardiology 670, University Hospital Nijmegen, P.O. Box 9101, 6500 HB, Nijmegen, The Netherlands

## Abstract

This case report describes about a young, male patient with persisting syncope during physical therapy for complex regional pain syndrome type 1 after metatarsal fractures.

The patient was referred to the Emergency Department, where Brugada syndrome was diagnosed. A cardioverter defibrillator was prophylactically implanted successfully. After this procedure, there were no contraindications for resuming further physical therapy for his painful foot. No clear causal inference with Brugada could be drawn from the complex regional pain syndrome type 1 or physical therapy described in this case report. Hyperthermia may, however, occur during such therapy, which is associated with dysrhythmia in general.

## Case presentation

### Background

Brugada syndrome is an autosomal dominant disease that can cause syncope and sudden cardiac death in young individuals with a normal heart [1].

It is characterized by an electrocardiographic pattern of complete or incomplete right bundle branch block and ST segment elevation in leads V1–V3. Around 20–25% of patients affected by this syndrome have genetic mutations in SCN5A, which cause a functional reduction in the availability of the cardiac sodium current in Brugada syndrome. The relative male preponderance of the phenotype, despite equal inheritance of the gene in males and females, has led to the speculation of a role for testosterone in the phenotype [2,3]. The disease may manifest first as cardiac arrest without any previous symptoms. The electrocardiographic pattern may be intermittent, requiring a pharmacological challenge with Class I anti-dysrhythmic drugs like flecainide to unmask the ST elevation. Dysrhythmias and sudden death generally occur during sleep or rest, but features of ECGs from Brugada patients also illustrate a cardiac sodium channel mutation. The arhythmogenicity characteristic of this mutation is revealed only at temperatures approaching the physiological range, and this suggests that some patients may be at greater risk during hyperthermia [4].

According to the International Association for the Study of Pain (IASP) in 1994, reflex sympathetic dystrophy and its many synonyms are collectively referred to as Complex Regional Pain Syndrome (CRPS). Two types can be distinguished: CRPS-1 may occur in an extremity after even a minor injury or operation, and CRPS-2 may occur only when nerve injury is involved. In the acute phase, signs and symptoms of inflammation or ischemia within the affected extremity characterize CRPS. Although the clinical signs and symptoms of CRPS are well known, the underlying pathophysiology remains unclear despite the knowledge that disuse precedes CRPS in most cases. Many clinicians use the Bruehl criteria to make a proper diagnosis, in which continuing pain disproportionate to the provocative event is the most frequent symptom [5]. Sensory, vasomotor, or sudomotor changes with edema, combined with a decreased range of motion with motor dysfunction and trophic changes, must also be present for diagnosing CRPS-1.

An accepted portion of the treatment consists of pain-contingent physical therapy to regain function and strength in the affected limb [6]. Pain Exposure Physical Therapy (PEP-therapy) is a different approach, in which functional physical therapy is combined with a cognitive – behavioral form of treatment for patients who are well-motivated to achieve functional targets. They are invited to use an active coping style to realize the treatment goal during five sessions spanning three months, and they are evaluated three months after the last session (time contingent). For this type of therapy, the use of analgesics, splints, and crutches is reduced. Additionally, treatment does not focus on the existing pain, and patients are instructed that pain should not be interpreted as a signal of tissue damage. This process disrupts the vicious circle of fear of movement, pain avoidance, and dysregulation of the sympathetic and central nervous system. Touching and moving the extremity decreases the fear of movement and increases the extremity's mobility. Clear treatment plans and empathic communication, as well as stimulation and positive feedback, are part of the standard plan. The patient is encouraged to attempt to achieve a realistic treatment goal for the next session by practicing the exercises at home. This approach has been shown to be successful in children [7]. No evidence has been provided yet for treating adult patients with CRPS-1 in this way, but we have been successfully treating patients with therapy-resistant CRPS-1 since 2005. A first report describing our results is in preparation.

The purpose of this case report is to describe a patient in whom Brugada syndrome was unmasked during PEP-therapy for CRPS-1 after metatarsal fractures. This report is intended to increase the awareness of healthcare workers, and to help recognizing the risk of strenuous physical therapy in such patients. Hypothetical causal inference should, however, always be interpreted with caution. An association of CRPS-1 with Brugada syndrome has never been reported. It is therefore more likely to assume a relationship with this patient's active physical therapy involvement during the presentation of his symptoms.

### Patient History

A 41-year old, slender, Caucasian male was referred to our outpatient department two years after having sustained midshaft fractures of the second and third metatarsal bone due to the crash of a heavy tent-pole. Both fractures were successfully treated non-operatively. The pain in his foot, however, did not resolve with normal physical therapy, and six months after the accident he presented with symptoms of a CRPS-1 according to the criteria of Bruehl [5]. He was referred to our hospital's department of physical therapy to undergo PEP-therapy in order to regain function of the lower extremity and be able to walk without restrictions.

He was otherwise healthy, and no co-morbidities or contra-indications were reported. He was not known with tobacco, drugs or alcohol abuse.

### Examination

This patient was seen in the department of physical therapy for his first treatment. Earlier treatment with pain contingent physical therapy, manual therapy, acupuncture, and action potential stimulation (APS) therapy had resulted in independent gait with orthopaedic shoes that were adapted to relieve his forefoot. The only pain medication he used was paracetamol. Pain relief and improvement of walking ability were the main reasons for which he sought treatment.

Physical examination showed a healthy, slender white male with a height of 193 cm and a weight of 80 kgs without further abnormalities. He presented with symptoms of allodynia, hyperesthesia, vasomotor, sudomotor and trophic changes, a decrease in the active and passive range of motion for the right ankle and toes, dystonia during foot extension, and a burning sensation during walking. He avoided placing any weight bearing on his forefoot and presented a passive way of walking.

After the painful examination, he was well motivated to begin treatment. His allodynia was treated with resumption of passive mobility during the first visit.

### Treatment

The means provided in Pain Exposure Physical therapy (PEP-therapy) are articular movement, including traction, translation, manipulation, massage, desensitization, flooding (experience of the most fearful action), muscle lengthening, and guiding. Exercising of the normal movement patterns (functional training) with posture and relaxation exercises in front of a mirror accompany instructions, information, and advice. The patient is taught to experience the sensation of pain as non-functional and practice the exercises at home. Because pain experience is rational and rational attention is redirected from pain behavior to the ability to move, the patient's pain behavior will decrease as his self-confidence in his physical abilities increases. This progress is encouraged by positive feedback from two therapists and the patient's partner.

Touching and massaging the painful foot was initiated with the explanation that the pain sensation is not harmful. The dystonic extensors of the foot were stretched, and the patient was instructed to do so himself. Touching and stretching caused a strong pain perception, which the patient expressed via facial grimaces and tightening of his muscles.

At the end of this first foot and ankle treatment session, the patient suddenly blacked out, recovered spontaneously, and began hyperventilating. According to his accompanying wife, these symptoms were common for him and hyperventilation had already occurred several years earlier. Because no one had asked, the patient had not mentioned this fact before. This time, however, the patient was still uneasy after an hour of relaxation and breath control exercises. The physical therapist advised him to attend the hospital's Emergency Ward to further evaluate hyperventilation with syncope [8].

### Outcome

This patient presented at the Emergency Ward with symptoms of blurred vision, tingling hands, and numbness of the right cheek. He had shown incidental hyperventilation throughout the last fifteen years. In one instance, he had collapsed when such an episode occurred while he was gardening. Cardiologic evaluation did not reveal any abnormalities at that time. He mentioned a recent sudden collapse in the shower with rapid spontaneous recovery without premonitory symptoms. He denied any other specific (cardiac) complaints such as chest pain, palpitations, or dyspnoea. His medical history was otherwise uneventful except for an inguinal hernia repair during childhood. A family history notes that his father survived a myocardial infarction at the age of fifty years. His fraternal twin brother had fully recovered from epilepsy during his early childhood. One younger brother died shortly after birth from severe spina bifida.

**Figure 1:**
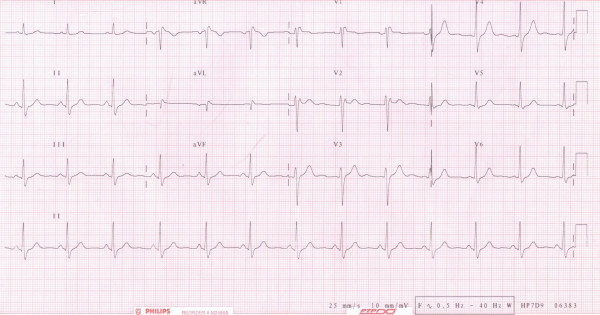
**Electrocardiography at first presentation**. Electrocardiography at first presentation with a high take-off ST segment elevation with upward concavity and positive or biphasic T-wave resulting in a saddleback configuration.

**Figure 2:**
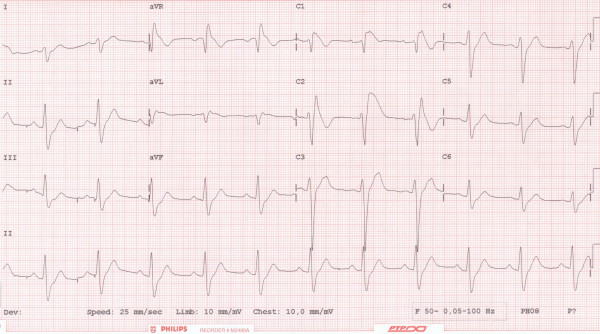
**Electrocardiography after provocation with flecainide**. Electrocardiography after provocation with flecainide with a shift from type II to type I.

**Figure 3:**
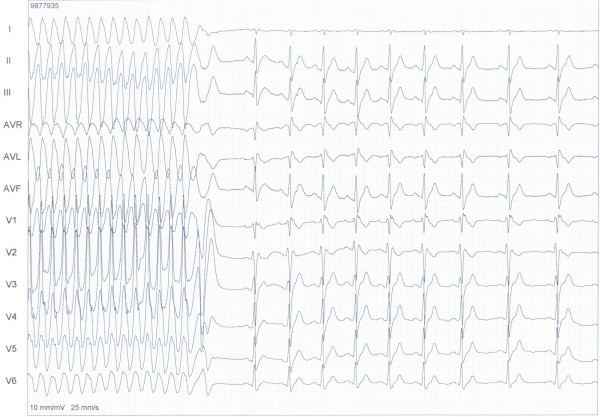
**Electrophysiologic study shows ventricular flutter**. Electrophysiologic study shows ventricular flutter after pacing the right ventricle with 500 min^-1^.

Electrocardiography showed a Brugada-like pattern with typical ST segment elevation in the right precordial leads, which has been designated as type II Brugada (fig. 1) [9]. He was admitted for further evaluation. A flecainide provocation test was positive, with a shift from type II to type I, accompanied by symptoms of syncope (fig. 2). Provocation during an electrophysiological study with pacing of the right ventricle produced a ventricular flutter with recognizable symptoms of syncope, which lasted seven seconds and terminated spontaneously (fig. 3). An automated implantable cardioverter defibrillator (AICD) was prophylactically implanted, and the patient's twin brother was advised to undergo screening.

Although no contraindications existed for resumption of the physical therapy according to recent guidelines, the patient preferred to accept the current condition of his painful foot [10].

## Discussion

Strenuous physical therapy may evoke a variety of involuntary patient reactions like hyperventilation. When patients are hyperventilating and do not recover spontaneously or respond to common treatment strategies like paper bag rebreathing, syncope may occur. Any syncope requires thorough clinical evaluation to exclude serious disorders like cardiac arrhythmia and epilepsy [8].

Brugada syndrome is a rare cardiac arrhythmia that may cause syncope in otherwise healthy young people. Several conditions producing Brugada-like electrocardiographic patterns, like the 'long QT syndrome,' should be borne in mind and excluded while making a diagnosis of the Brugada syndrome [1]. Treatment of patients suffering from Brugada is difficult, because pharmacological agents are not universally effective. State-of-the-art management involves the implantation of a cardioverter defibrillator. Additionally, symptomatic patients with inducible ventricular dysrhythmias and a positive family history, like the patient described in this report, should be considered for prophylactic implantation of a cardioverter defibrillator. This patient had a history of hyperventilation, which could have been a symptom of masked Brugada. However, hyperventilation may also be the cause of syncope. In addition, this may cause variant angina, which may in turn induce ischemic, life-threatening dysrhythmia. Our patient underwent painful PEP-therapy, which can be considered as strenuous exercise. Strenuous activities and sudden death have also lead to the identification of Brugada syndrome in athletes [1]. It has been assumed that strenuous exercise and its resultant increase in body core temperature can unmask the Brugada syndrome. This could have occurred during PEP-therapy for CRPS-1 in our patient [4].

The patient did not mention any form of hyperventilation during our first contact. Due to the presence of his wife, he remembered his history of hyperventilation and syncope during the treatment session. No association between Brugada syndrome and any form of physical therapy or CRPS-1 has ever been described. Therefore, any unexpected symptoms of syncope during strenuous or painful treatments deserve further evaluation to prevent sudden death and must be interpreted as a "red flag" or serious warning sign. Most physical therapists are adequately equipped with professional knowledge to detect these warning signs in patients undergoing treatments, such as the case demonstrated in this case report.

## Conclusion

Strenuous physical therapy may evoke a variety of involuntary patient reactions like hyperventilation. When patients are hyperventilating and do not recover spontaneously or respond to common treatment strategies like paper bag rebreathing, syncope may occur. Any symptoms of syncope during strenuous treatment or exercise deserve further evaluation to prevent sudden death.

## Abbreviations

CRPS: Complex Regional Pain Syndrome Type 1; ECG: Electrocardiogram; SCN5A: Sodium Channel voltage-gated type V alpha subunit; IASP: International Association for the Study of Pain; PEP-therapy: Pain Exposure Physical-therapy; AICD: Automated Implantable Cardioverter Defibrillator.

## Consent

Written informed consent was obtained from the patient for publication of this case report and any accompanying images. A copy of the written consent is available for review by the Editor-in-Chief of this journal.

## Competing interests

The authors declare that they have no competing interests.

## Authors' contributions

JF did the initial clinical assessment of the patient and drafted the manuscript. AB wrote the part about CRPS diagnosis. FK treated the patient and wrote the part about CRPS treatment

JS treated the patient and corrected the part about Brugada. All authors read and approved the final manuscript.
